# SARS-CoV-2 genomic variations associated with mortality rate of COVID-19

**DOI:** 10.1038/s10038-020-0808-9

**Published:** 2020-07-22

**Authors:** Yujiro Toyoshima, Kensaku Nemoto, Saki Matsumoto, Yusuke Nakamura, Kazuma Kiyotani

**Affiliations:** Project for Immunogenomics, Cancer Precision Medicine Center, Japanese Foundation for Cancer Research, Tokyo, 135-8550 Japan

**Keywords:** Immunogenetics, Infection

## Abstract

The coronavirus disease 2019 (COVID-19) outbreak, caused by SARS-CoV-2, has rapidly expanded to a global pandemic. However, numbers of infected cases, deaths, and mortality rates related to COVID-19 vary from country to country. Although many studies were conducted, the reasons of these differences have not been clarified. In this study, we comprehensively investigated 12,343 SARS-CoV-2 genome sequences isolated from patients/individuals in six geographic areas and identified a total of 1234 mutations by comparing with the reference SARS-CoV-2 sequence. Through a hierarchical clustering based on the mutant frequencies, we classified the 28 countries into three clusters showing different fatality rates of COVID-19. In correlation analyses, we identified that ORF1ab 4715L and S protein 614G variants, which are in a strong linkage disequilibrium, showed significant positive correlations with fatality rates (*r* = 0.41, *P* = 0.029 and *r* = 0.43, *P* = 0.022, respectively). We found that BCG-vaccination status significantly associated with the fatality rates as well as number of infected cases. In BCG-vaccinated countries, the frequency of the S 614G variant had a trend of association with the higher fatality rate. We also found that the frequency of several *HLA* alleles, including HLA-A*11:01, were significantly associated with the fatality rates, although these factors were associated with number of infected cases and not an independent factor to affect fatality rate in each country. Our findings suggest that SARS-CoV-2 mutations as well as BCG-vaccination status and a host genetic factor, *HLA* genotypes might affect the susceptibility to SARS-CoV-2 infection or severity of COVID-19.

## Introduction

The novel betacoronavirus, severe acute respiratory syndrome coronavirus 2 (SARS-CoV-2), which causes coronavirus disease 2019 (COVID-19), was first reported in Wuhan, China in December 2019 [1, 2]. Soon after, the virus caused an outbreak in China and has spread to the world. According to the World Health Organization, the current outbreak of COVID-19 has nearly 11.5 million confirmed cases worldwide with more than 530,000 deaths, as of July 6, 2020. The SARS-CoV-2 genome comprises of around 30,000 nucleotides organized into specific genes encoding structural proteins and nonstructural proteins (Nsps) [1, 2]. Structural proteins include spike (S), envelope (E), membrane (M), and nucleocapsid (N) proteins. Surface S glycoprotein is involved in the interaction with the host’s angiotensin-converting enzyme 2 (ACE2) receptor and plays an important role in rapid human to human transmission. Nsps, which are generated as cleavage products of the open reading frame 1ab (ORF1ab) viral polyproteins, assemble to facilitate viral replication and transcription. RNA-dependent RNA polymerase, also known as Nsp12, is the key component that regulates viral RNA synthesis with the assistance of Nsp7 and Nsp8 [3]. In addition, five accessory proteins are encoded by ORF3a, ORF6, ORF7a ORF8, and ORF10 genes.

SARS-CoV-2 has rapidly spread around the world compared with SARS-CoV appeared in 2002 and Middle East respiratory syndrome coronavirus (MERS-CoV) in 2012. Although the estimated fatality rate in the confirmed cases is 6.6% in SARS-CoV-2, which is lower than those of SARS-CoV and MERS-CoV, 9.6% and 34.3%, respectively [4], there is an urgent need for its effective treatment based on antivirals and vaccines that reduce the mortality and morbidity rates of COVID-19. However, up to now, the causes of the large country-by-country difference of the mortality rates related to COVID-19 have not been clearly understood. Although many studies were conducted, the effects of SARS-CoV-2 genetic variations and host genetic factors remain elusive.

In this study, we comprehensively analyzed 12,343 SARS-CoV-2 genome sequences isolated from patients/individuals in six geographic areas, including Asia, North America, South America, Europe, Oceania, and Africa, and investigated their correlations to the fatality rates in 28 different countries. We also investigated the associations with BCG-vaccination status as well as human leukocyte antigen (HLA), which is an important molecule to recognize virus by our host immune system.

## Methods

### Coronavirus sequences

Full-length viral nucleotide sequence of the reference SARS-CoV-2 (accession number MN908947) [1] was downloaded from the NCBI GenBank. We used a total of 12,343 SARS-CoV-2 sequences isolated in 50 different countries of six geographic areas, including 1062 sequences from Asia, 4060 from North America, 99 from South America, 6012 from Europe, 1028 from Oceania, and 82 from Africa regions, which were deposited in the Global Initiative on Sharing Avian Influenza Data as of 7 May 2020 [5]. To analyze mutations based on countries, we used the data of 28 countries in which more than 30 SARS-CoV-2 sequences are available, among the 50 countries.

### Mutation analysis

We analyzed mutations of SARS-CoV-2 as described previously [6]. Briefly, we first aligned each of the SARS-CoV-2 sequences to the reference sequence SARS-CoV-2_Wuhan-Hu-1 (accession number MN908947) using BLAT software [7]. After the alignment, we extracted nucleotide sequences corresponding to individual proteins of SARS-CoV-2, translated them into amino acid sequences, and then compared them to reference amino acid sequences of SARS-CoV-2_Wuhan-Hu-1 (accession numbers QHD43415-QHD43423, QHI42199).

### Data acquisition

Data on numbers of confirmed cases and deaths related to COVID-19 were obtained from the Worldometer (https://www.worldometers.info/coronavirus/) on 7 May 2020 (Supplementary Table 1). Data of confirmed cases and deaths in each state in the United States were obtained on 3 July 2020. Fatality rate in infected individuals was calculated from total infected cases and total deaths in each country. The allelic frequencies of *HLA* genes were obtained from The Allele Frequency Net Database [8]. Data on BCG-vaccination status in each country were obtained from the previous reports [9–11].

### Statistical analyses

Continuous variables were compared using the Student’s *t* test. Fisher’s exact test was used to analyze differences of mutation rates of SARS-CoV-2 among the different geographic areas. A hierarchical clustering was performed to identify clusters corresponding to distinct subgroups with the selected mutations using R package stats. Global maps of clusters or mutations were drawn using R package rworldmap. Pearson’s correlation was used to evaluate correlations among mutant frequencies, *HLA* allele frequencies and fatality rates. Haploview software was used to analyze and visualize the haplotypes of SARS-CoV-2 mutations [12]. Multiple regression analysis was used to test for an independent contribution of identified factors to fatality rates of COVID-19. All statistical analyses were carried out using the R statistical environment version 3.6.1.

## Results

**Fig. 1:**
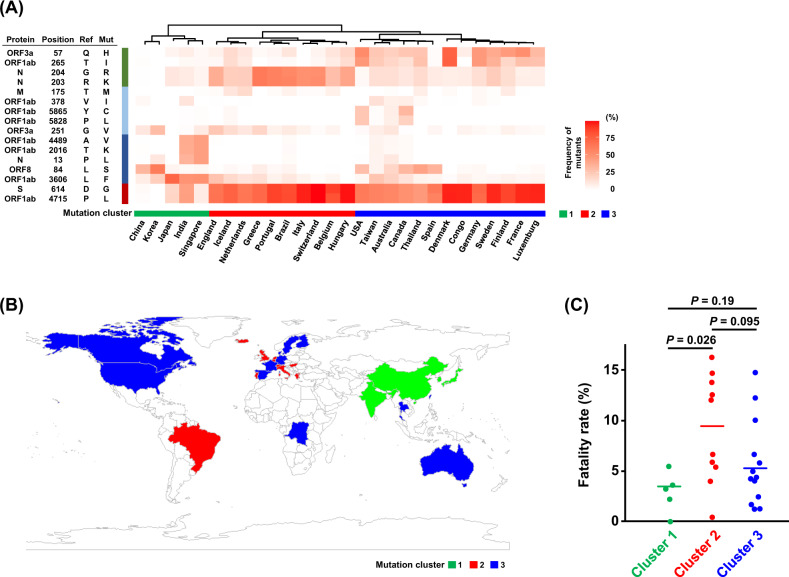
Clustering analysis of SARS-CoV-2 among 28 countries. **a** Heatmap for the frequencies of SARS-CoV-2 mutants. The 28 countries were classified into three clusters based on the mutational signature by a hierarchical clustering. Protein sequence based on the SARS-CoV-2_Wuhan-Hu-1 sequence (GenBank accession number MN908947) is used as a reference. Ref; amino acid in reference SARS-CoV-2 sequence, Mut, amino acid in mutant SARS-CoV-2. **b** A global mapping of the three clusters. **c** Fatality rates according to the clusters. Horizontal lines represent the means. The Student’s *t* test was used to evaluate statistical significance

All replicating viruses, including coronavirus, continuously accumulate genomic mutations that persist due to natural selections. These mutations contribute to enhancement of ability of viral proliferation and infection as well as an escape from host immune attack. We firstly investigated mutations in 12,343 SARS-CoV-2 genome sequences isolated from patients/individuals in six different regions, including Asia, North America, South America, Europe, Oceania, and Africa. We identified a total of 1234 mutations detected in at least two independent samples, including 131 mutations found at a frequency of more than 10% (Supplementary Table 2). A hierarchical clustering using 16 common amino acid mutations classified 28 countries into three clusters (Fig. 1a). The cluster 1 includes most of the Asian countries we analyzed, whereas the cluster 2 includes European and South American countries, and the cluster 3 includes European, North American, Oceania, African and a few Asian countries (Fig. 1b). Comparing the mutations among the three clusters, the average frequency of an L variant of an ORF1ab P4715L in the countries classified as the cluster 1 was 14.7%, which is significantly lower than 81.3% and 73.2%, respectively, in the countries classified as the clusters 2 and 3 (*P* = 1.3 × 10^−6^ and *P* = 2.5 × 10^−5^, respectively; Supplementary Fig. 1A). The ORF1ab 4715L variant was detected at the significantly low frequency in Asian countries compared with the other areas (20.8% vs. others 54.9–86.8%, *P* = 1.1 × 10^−118^; Supplementary Fig. 2). Similarly, the frequency of a G variant of S protein D614G was significantly lower in the cluster 1 than the other two clusters (*P* = 1.2 × 10^−6^ and *P* = 1.7 × 10^−5^, respectively, for the clusters 2 and 3; Supplementary Fig. 1B). In the cluster 2, K/R variants of N protein R203K/G204R mutations were significantly enriched at 43.1%, compared with the other clusters (5.2%, *P* = 0.00011 for the cluster 1 and 11.8%, *P* = 5.6 × 10^−7^ for the cluster 3; Supplementary Fig. 1C). In addition, in the cluster 1, L and F variants of N P13L and ORF1ab L3606F were predominantly enriched. The L variant of N P13L was found at 17.8%, which was significantly higher than 0.2% and 1.4%, respectively, in the clusters 2 and 3 (*P* = 0.012 and *P* = 0.0079; Supplementary Fig. 1D). The F variant of ORF1ab L3606F was detected at a higher frequency of 40.1% than 10.0% and 7.9% in the clusters 2 and 3, respectively (*P* = 0.0035 and *P* = 0.00050; Supplementary Fig. 1E). To further analyze the mutational profile, we performed a haplotype analysis by drawing a linkage disequilibrium (LD) map for SARS-CoV-2 viral genomes (Supplementary Fig. 3). We found that ORF1ab 4715L and S protein 614G variants were in a nearly complete LD (*r*^2^ of LD = 0.98 and *D’* = 1.00). N protein 203K/204R variants were additionally acquired in the S protein 614G type of virus genome as indicated as *r*^2^ of LD = 0.11 and *D’* = 0.99. These results indicate that S protein 614G-N protein 203K/204R haplotype characterizes the cluster 2.

**Fig. 2:**
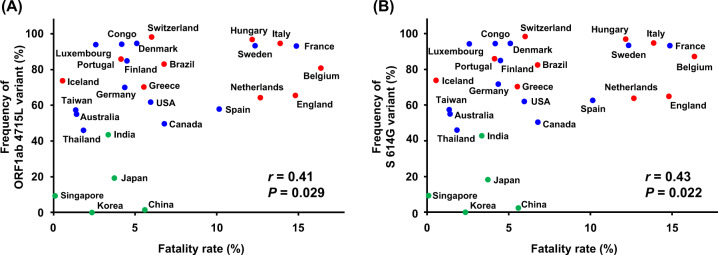
Correlation analysis of variant frequencies of SARS-CoV-2 ORF1ab 4715L (**a**) or S 614G (**b**) with fatality rates of COVID-19 among 28 countries. Pearson’s correlation coefficients (*r*) were calculated. Colors of each dot were corresponding to the mutational clusters shown in Fig. 1a

We then investigated the association with the fatality rates among confirmed cases in the 28 countries. In the analysis comparing the fatality rates in the countries classified as either of the three clusters, average fatality rate of the countries belonging to the cluster 2 was 9.3%, which was higher than 3.0% and 5.8% of averages of the countries belonging to the clusters 1 and 3, respectively (*P* = 0.026 and *P* = 0.095; Fig. 1c). Among the mutations we analyzed, the frequencies of ORF1ab 4715L-type and S 614G-type viruses showed significant positive correlations with fatality rates (Pearson’s correlation coefficient (*r*) = 0.41, *P* = 0.029 and *r* = 0.43, *P* = 0.022, respectively; Fig. 2a, b). Since the clusters 2 and 3 were separated mainly by the frequency of N 203K/204R, we also examined the correlations of this variant or S 614G-N 203R/204G haplotype with fatality rates; however, the correlations were not statistically significant (*r* = 0.31, *P* = 0.11; *r* = 0.27, *P* = 0.17, respectively; Supplementary Fig. 4A, B).

**Fig. 3:**
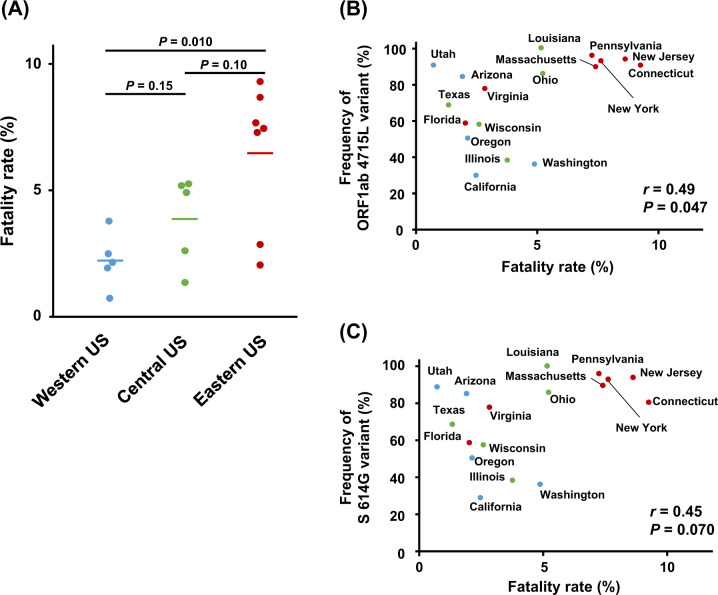
Association of variant frequencies of SARS-CoV-2 with fatality rates of COVID-19 among 17 states in the United States. **a** Fatality rates in three different areas in the United States, Western, Central, and Eastern. Horizontal lines represent the means. The Student’s *t* test was used to evaluate statistical significance. **b**, **c** Correlation analysis between frequencies of SARS-CoV-2 ORF1ab 4715L (**b**) or S 614G variants (**c**) and fatality rates. Pearson’s correlation coefficients (*r*) were calculated

It is reported that fatality rates are different among the areas or states in the United States [13]. When we compared fatality rates among the three different areas, Western, Central and Eastern, in the United States, an Eastern area showed a higher fatality rate of 6.5% than that of 2.2% in a Western area (*P* = 0.010) and that of 3.9% in a Central area (*P* = 0.10; Fig. 3a). Therefore, we further investigated the correlations of the variants with fatality rates in the 17 states. The frequencies of ORF1ab 4715L- and S protein 614G-types tended to show positive correlations with the fatality rates (*r* = 0.49, *P* = 0.047; *r* = 0.45, *P* = 0.070, respectively; Fig. 3b, c). Even when integrating the data of 17 states and the remaining 27 countries, the significant correlations kept significant (*r* = 0.38, *P* = 0.014; *r* = 0.39, *P* = 0.011, respectively; Supplementary Fig. 5A, B).

**Fig. 4:**
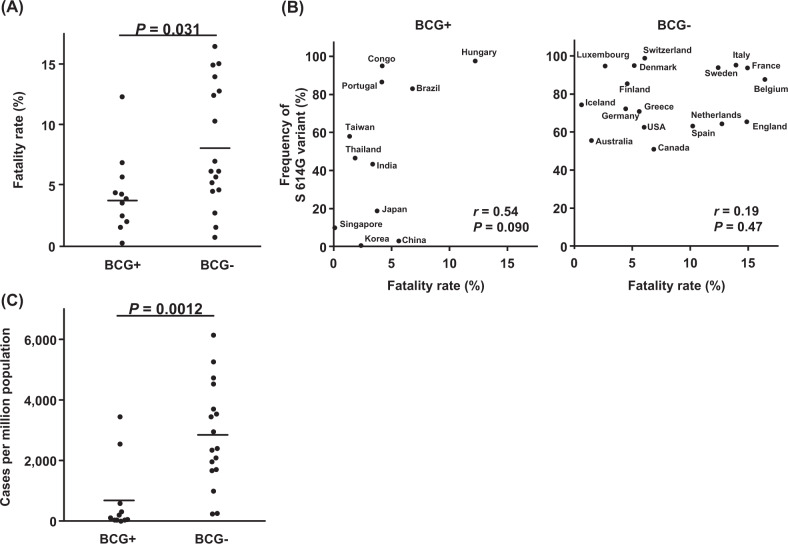
Association of BCG-vaccination status with fatality rates and infected cases of COVID-19 among 28 countries. **a** Fatality rates in BCG-vaccinated (BCG+) and BCG-non-vaccinated countries (BCG−). Horizontal lines represent the means. The Student’s *t* test was used to evaluate statistical significance. **b** Correlation analysis between frequencies of S 614G variant of SARS-CoV-2 and fatality rates in BCG+ and BCG− countries. Pearson’s correlation coefficients (*r*) were calculated. **c** Number of infected cases in BCG+ and BCG− countries. Horizontal lines represent the means. The Student’s *t* test was used to evaluate statistical significance

Several other factors are investigated in association with mortality related to COVID-19. Ecological studies have suggested that countries that mandate BCG vaccination for the population have a lower number of infections and a reduced mortality from COVID-19, although the association is still controversial and the underlying mechanism has not been clarified [9, 14, 15]. We classified 28 countries into two groups according to the BCG-vaccination status as the routine vaccine schedules. As a result, the mean of fatality rates was significantly lower in 11 BCG-vaccinated countries than in 17 BCG-non-vaccinated countries (4.1% vs. 8.1%, *P* = 0.031; Fig. 4a). When we divided BCG-vaccinated countries into subgroups according to the strains of BCG vaccine, we observed some differences in the fatality rates among the countries by different strains of BCG vaccine, but sample sizes of subgroups are too small to evaluate statistical significance (Supplementary Fig. 6). We also found the frequencies of S 614G variant showed a trend of positive correlation with fatality rates (*r* = 0.54, *P* = 0.090; Fig. 4b) in BCG-vaccinated countries, but such correlation was not observed in BCG-non-vaccinated countries (*r* = 0.19, *P* = 0.47; Fig. 4b). In addition, the number of confirmed cases per million population was significantly lower in BCG-vaccinated countries than in BCG-non-vaccinated countries (710 vs. 2912, *P* = 0.0012; Fig. 4c). These results suggest that BCG-vaccination may protect from SARS-CoV-2 infection by potentiation of innate immune response; however, ORF1ab 4715L-type and S protein 614G-type SARS-CoV-2 variants may escape from the immune response.

**Fig. 5:**
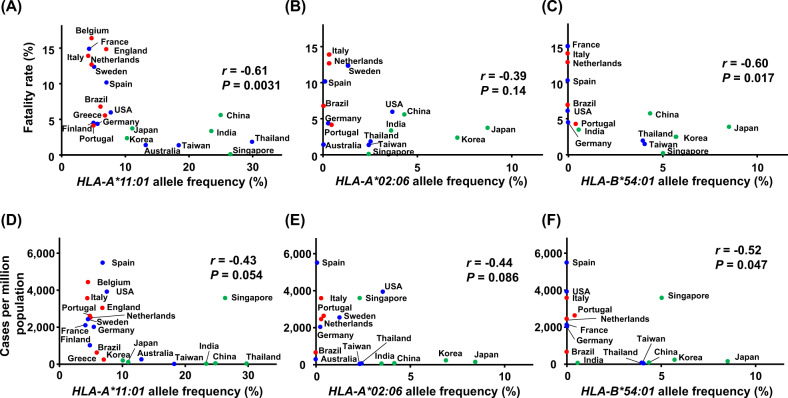
Association of *HLA* allele frequency with fatality rates and infected cases of COVID-19 among countries. **a**–**c** Correlation between HLA-A*11:01 (**a**), HLA-A*02:06 (**b**), and HLA-B*54:01 (**c**) allelic frequencies and fatality rates of COVID-19. Numbers of analyzed countries are 21, 16, and 15, respectively, for HLA-A*11:01, HLA-A*02:06, and HLA-B*54:01. Pearson’s correlation coefficient (*r*) was calculated. **d**–**f** Correlation between HLA-A*11:01 (**d**), HLA-A*02:06 (**e**), and HLA-B*54:01 (**f**) allelic frequency and number of infected cases of COVID-19. Pearson’s correlation coefficient (*r*) was calculated

Host genetic differences, especially in *HLA* loci, are well-known to contribute to individual variations in the immune responses to pathogens. We finally searched peptide epitopes with a high binding affinity to HLA molecules, which we previously reported [6], involving the two SARS-CoV-2 mutations, ORF1ab P4715L and S D614G, to investigate the association with host immune responses. We found that several epitopes, which include the position of ORF1ab P4715L or S protein D614G, are possibly bind to HLA molecules, including HLA-A*02:06, HLA-A*11:01, HLA-B*07:02, and HLA-B*54:01, although the mutated epitopes from variant SARS-CoV-2 also predicted to bind to HLA molecules at similar affinities (Supplementary Table 3). Using the information of 21 countries in which allele frequency data are available, we examined a relationship between allele frequency of HLA-A*11:01 and the fatality rates. Consequently, we found a significant negative correlation (*r* = −0.61, *P* = 0.0031; Fig. 5a). Similarly, a trend of negative correlations was observed between allele frequencies of HLA-A*02:06 or HLA-B*54:01 and the fatality rates (*r* = −0.39, *P* = 0.14, *N* = 16 and *r* = −0.60, *P* = 0.017, *N* = 15; Fig. 5b, c). However, the significant correlations became not statistically significant after adjusted by the frequency of S 614G variant in multiple regression (*P* = 0.13 for HLA-A*11:01, *P* = 0.73 for HLA-A*02:06 and *P* = 0.45 for HLA-B*54:01). We also found negative correlations between allele frequencies of the *HLAs* and the number of confirmed cases per million population (*r* = −0.43, *P* = 0.054 for HLA-A*11:01, r = −0.44, *P* = 0.086 for HLA-A*02:06 and *r* = −0.52, *P* = 0.047 for HLA-B*54:01; Fig. 5d–f). Together, these results suggest that differences in *HLA* allele frequencies may explain different susceptibilities to SARS-CoV-2 infection among the countries, although there are many other potential confounding factors needed to be considered.

## Discussion

The current outbreak of COVID-19 has rapidly spread worldwide. Most patients with COVID-19 exhibit no or mild to moderate symptoms, but ~15% progress to severe pneumonia and about 5% eventually develop acute respiratory distress syndrome, septic shock, and multiple organ failures. The mortality rates related to COVID-19 vary among countries, generally known to be significantly higher in European and North American countries than those of Asian countries. Although several possibilities to explain the differences in the mortality rates are demonstrated, including the difference of age distribution, BCG-vaccination status, virus genomic types, and genetic backgrounds, nothing is clear at this moment. In this study, we investigated the SARS-CoV-2 virus mutations and found that the frequencies of S protein 614G variant and its highly linked variant, ORF1ab 4715L, were significantly correlated with fatality rates in the 28 countries and 17 states of the United States.

The D614G spike mutation is the mutation detected in Europe in the early phase and has widely spread around the globe, especially to European and North American countries [16–19]. Spike glycoprotein is essential for interaction with ACE2 expressed in host cells and is important for viral transmission [20, 21]. Therefore, spike glycoprotein is most vital hotspot of amino acid mutations when viruses acquire mutations to enhance the virus-cell entry to adapt environments. Structural analyses indicated that S protein having a D614G substitution is located on the surface of the virus and interacts with ACE2. Concordant to our results, a few reports demonstrated that S 614G variant was associated with the mortality related to COVID-19 [13, 22]. ORF1ab P4715L is located in Nsp12, which is important for viral RNA replication. We found significant associations between these mutations and the fatality rates; however, the functional significance of these mutations has not clarified yet.

Since immune responses through HLA and T cells are important to protect from virus infections and also known to be involved in the progression of COVID-19, we screened epitopes around the mutations associated with fatality rates (Supplementary Table 3). ORF1ab P4715L is located in the epitope sequences of ORF1ab 4713–4721, FPPTSFGPL, ORF1ab 4713–4722, FPPTSFGPLV, and ORF1ab 4715–4724, PTSFGPLVRK, which were predicted to have strong binding affinities of 44, 41, and 45 nM to HLA-B*07:02, HLA-B*54:01, and HLA-A*11:01, respectively. In a computational prediction, corresponding mutated peptides show higher binding affinities of 11, 12, and 23 nM. Similarly, S D614G is located in the epitope sequences of S606-615, NQVAVLYQDV, and S612-620, YQDVNCTEV. Both of wild-type and mutated epitopes were predicted to bind to HLA-A*02:06 at similar affinities. Among them, the countries where the proportion of individuals with HLA-A*11:01, HLA-A*02:06, and HLA-B*54:01 alleles are relatively high showed lower fatality rates as well as number of confirmed cases (Fig. 5). However, the significant correlations with fatality rates became not significant after adjusted by the frequency of S protein 614G-type virus in multiple regression analysis. These results suggest that individuals with HLA-A*11:01, HLA-A*02:06, or HLA-B*54:01 might be protected from infection of SARS-CoV-2, although further studies are needed to investigate the effects of other potential confounding factors, such as different phases of outbreak, age of infected population, management of the pandemic. In SARS-CoV and MERS-CoV, several *HLA* genotypes have been reported to associate with susceptibility or resistance, including HLA-B*07:03, HLA-B*46:01, HLA-C*08:01, HLA-C*15:02, HLA-DRB1*03:01, HLA-DRB1*11:01, and HLA-DRB1*12:02 [23–26]. Although further studies are required to elucidate whether such cytotoxic T lymphocytes targeting the epitopes are present in peripheral blood in patients, especially in severe patients, and also large scale case-control association studies are needed to confirm the association of *HLA* genotype with susceptibility or disease progression of SARS-CoV-2 infection, these findings in the current study provide an important insight into treatment of the current SARS-CoV-2 and prevention of the second SARS-CoV-2 pandemic.

In summary, we comprehensively investigated SARS-CoV-2 genome mutations, BCG-vaccination status, and *HLA* genotypes in the 28 different countries and identified significant associations of some virus genome variants with the fatality rates. These results may explain, at least a part of the differences of the SARS-CoV-2 infection or the mortality rates related to COVID-19 among various countries.

## Supplementary information

Supplementary Figure 1-6

Supplementary Table 1

Supplementary Table 2

Supplementary Table 3
