# Supplementary material for: SARS-CoV-2 genomic variations associated with mortality rate of COVID-19
Source: J Hum Genet. 2020 Jul 22;65(12):1075–82. doi: 10.1038/s10038-020-0808-9 (PMC7375454; doi:10.1038/s10038-020-0808-9)
Supplement: Supplementary file 1 — Supplementary Figure 1-6 [file 10038_2020_808_MOESM1_ESM.pdf]

Supplementary Data for

SARS-CoV-2 genomic variations associated with mortality rate of COVID-19

Yujiro Toyoshima[1], Kensaku Nemoto[1], Saki Matsumoto[1], Yusuke Nakamura[1], Kazuma Kiyotani[1]

[1]Project for Immunogenomics, Cancer Precision Medicine Center, Japanese Foundation for Cancer Research, Tokyo 135-8550, Japan

Index of Supplementary Data

Supplementary Figure 1. Frequencies of SARS-CoV-2 variants among the three clusters.

Supplementary Figure 2. Global maps for the frequencies of SARS-CoV-2 variants.

Supplementary Figure 3. Linkage disequilibrium (LD) map of mutations in 12,266 SARS-CoV-2 sequences.

Supplementary Figure 4. Correlation analysis of frequency of SARS-CoV-2 N 203K/204R variant (A) or haplotype frequency of S 614G/N 203R/204G (B) with fatality rates of COVID-19 among 28 countries.

Supplementary Figure 5. Correlation analysis of frequencies of SARS-CoV-2 ORF1ab 4715L (A) or S 614G variants (B) with fatality rates of COVID-19 among 27 countries and 17 states in the United States.

Supplementary Figure 6. Association of the strains of BCG vaccine with fatality rates of COVID-19 among 28 countries.

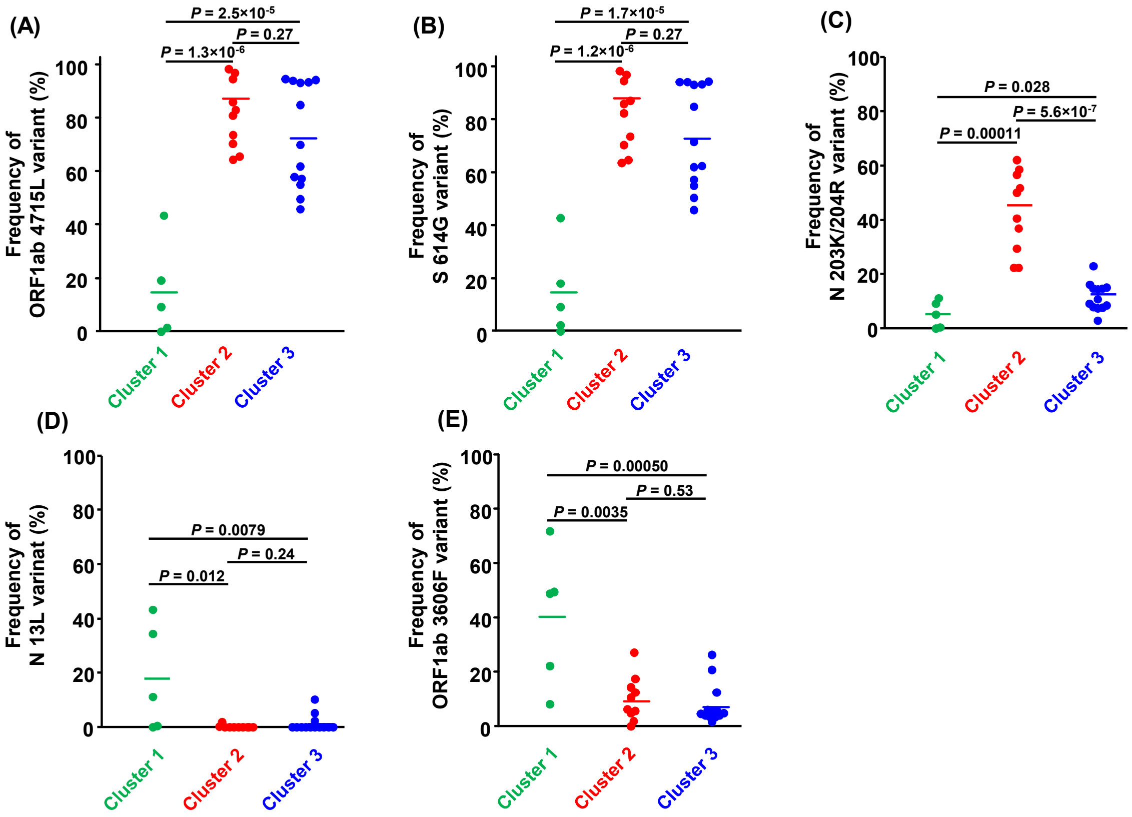

Supplementary Figure 1. Frequencies of SARS-CoV-2 variants among the three clusters. (A) ORF1ab 4715L, (B) S 614G, (C) N 203K/204R, (D) N 13L, (E) ORF1ab 3606F. Horizontal lines represent the means. The Student's *t* test was used to evaluate statistical significance. Color of each dot was corresponding to the mutational clusters shown in Fig. 1A.

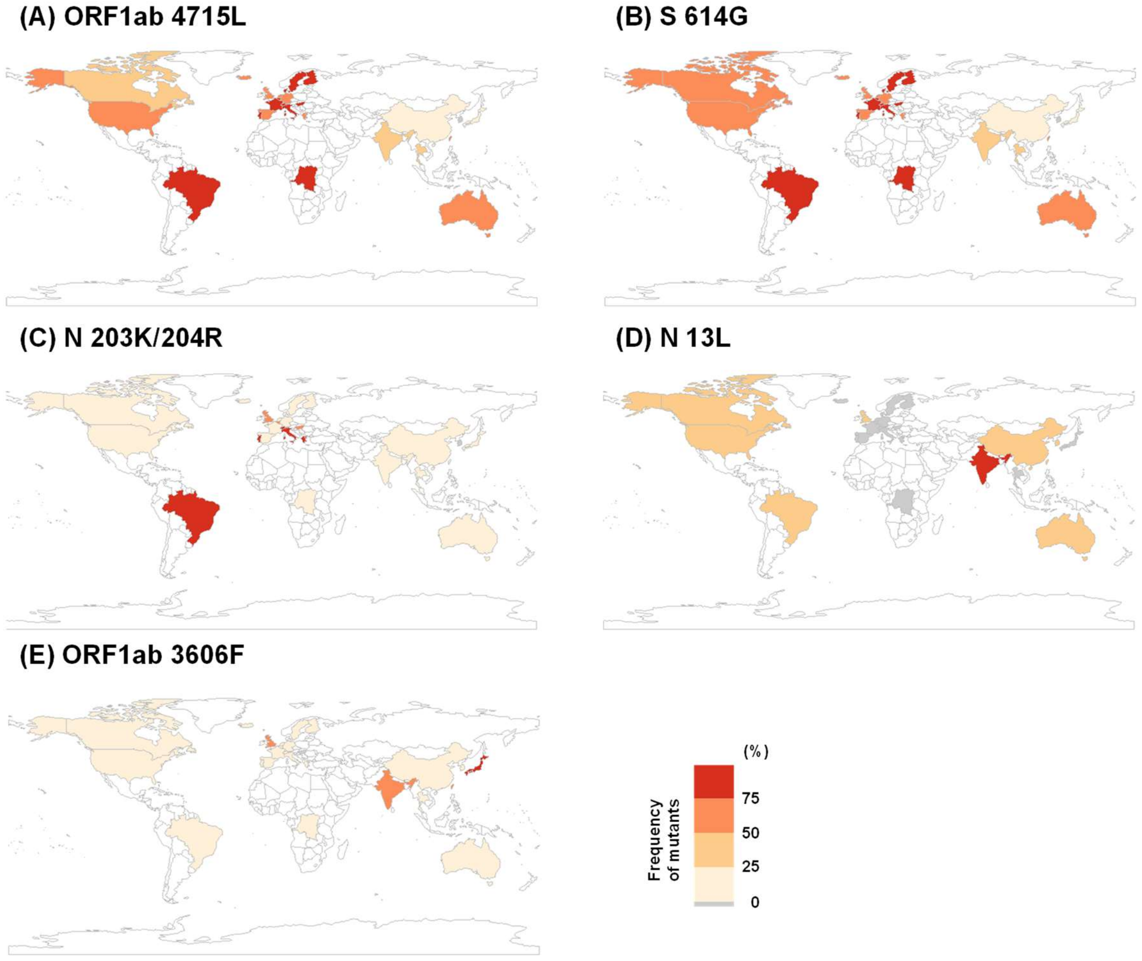

Supplementary Figure 2. Global maps for the frequencies of SARS-CoV-2 variants. (A) ORF1ab 4715L, (B) S 614G, (C) N 203K/204R, (D) N 13L, (E) ORF1ab 3606F.

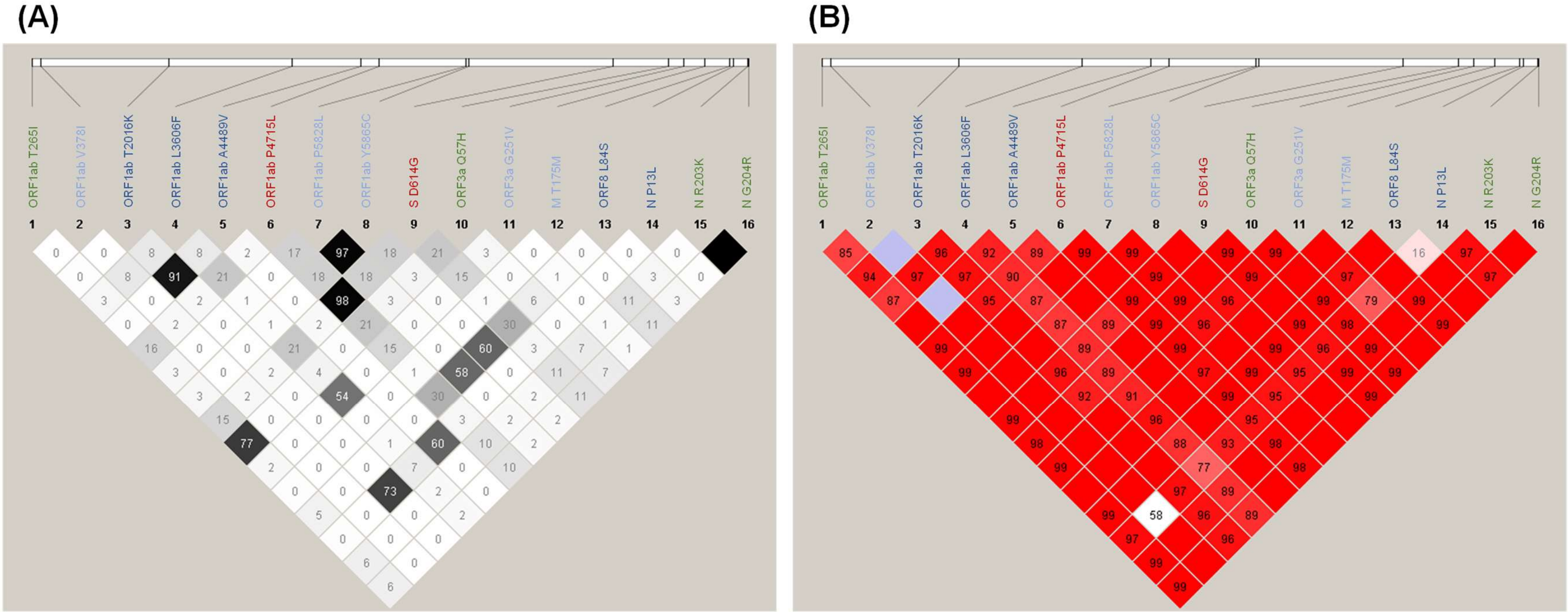

Supplementary Figure 3. Linkage disequilibrium (LD) map of mutations in 12,266 SARS-CoV-2 sequences. (A) r^2-based and (B) *D'*-based LD maps were drawn for 16 mutations, which were found at 10% in at least 2 of the 28 countries.

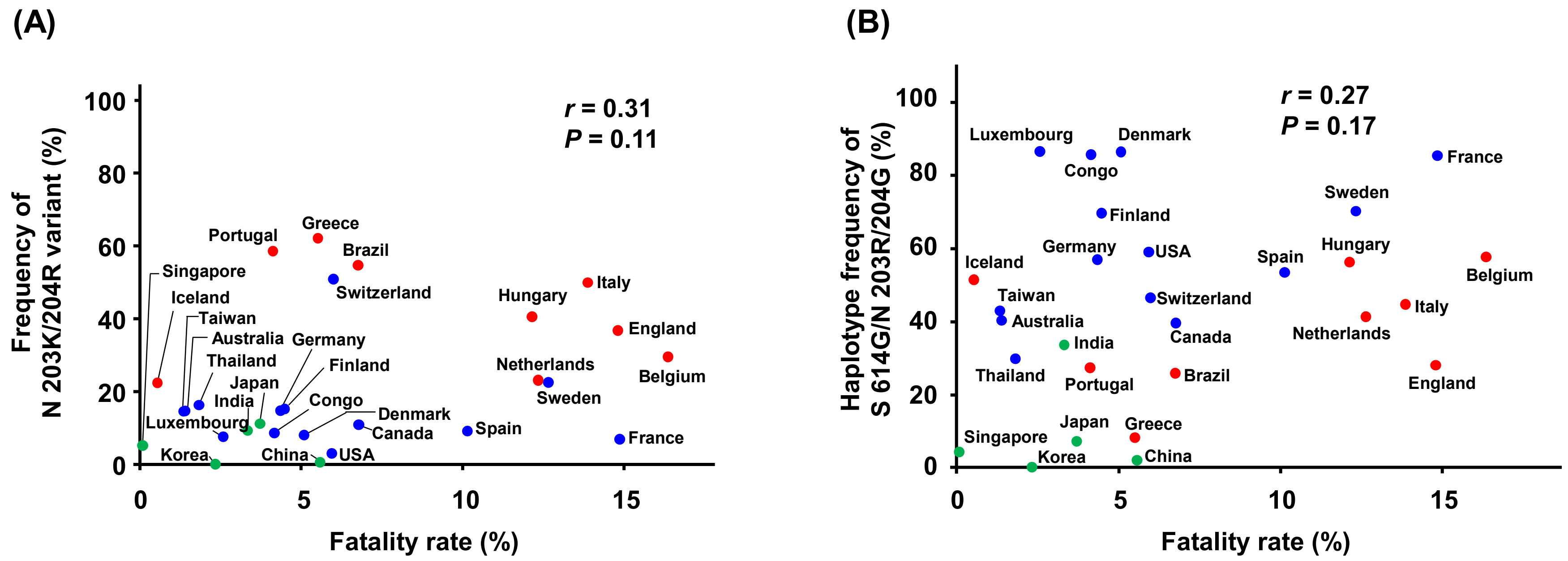

Supplementary Figure 4. Correlation analysis of frequency of SARS-CoV-2 N 203K/204R variant (A) or haplotype frequency of S 614G/N 203R/204G (B) with fatality rates of COVID-19 among 28 countries. Pearson's correlation coefficients (*r*) were calculated. Colors of each dot were corresponding to the mutational clusters shown in Fig. 1A

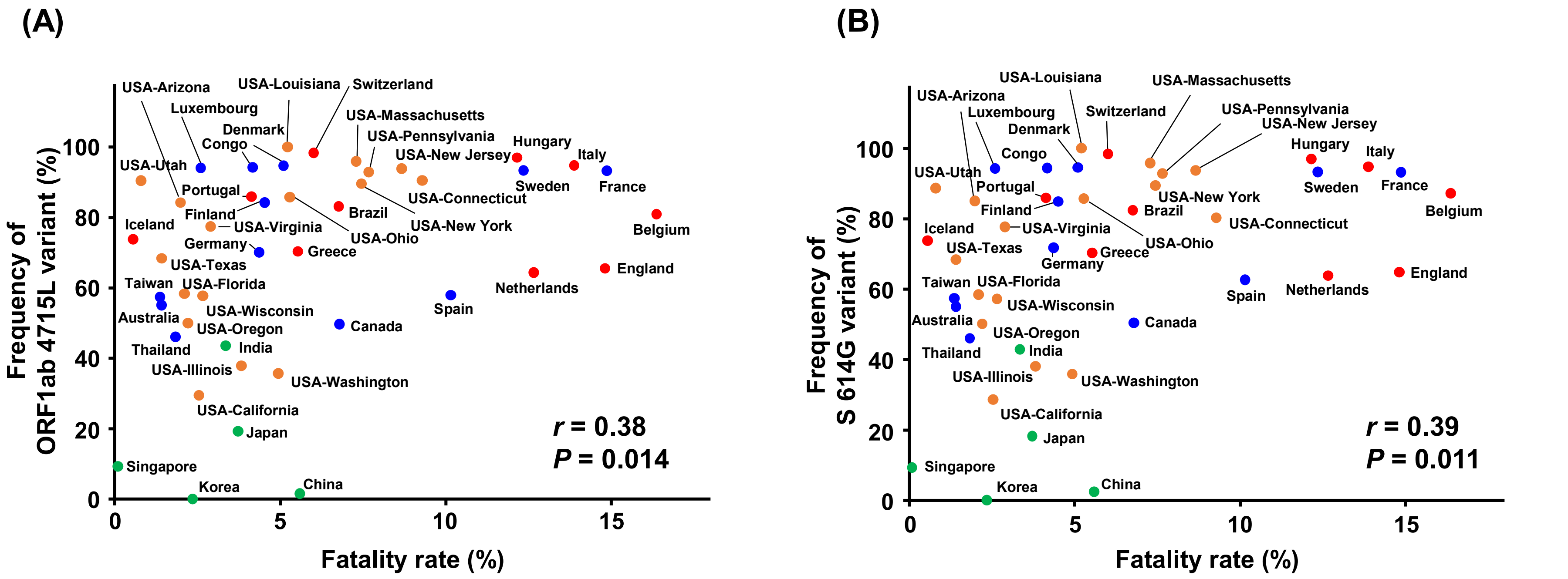

Supplementary Figure 5. Correlation analysis of frequencies of SARS-CoV-2 ORF1ab 4715L (A) or S 614G variants (B) with fatality rates of COVID-19 among 27 countries and 17 states in United States. Pearson's correlation coefficients (*r*) were calculated. Colors of each dot were corresponding to the mutational clusters shown in Fig. 1A.

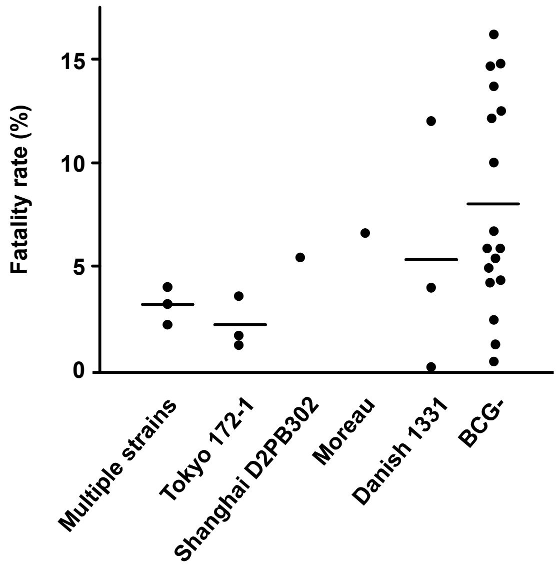

Supplementary Figure 6. Association of the strains of BCG vaccine with fatality rates of COVID-19 among 28 countries. Fatality rates in the countries using different strains of BCG vaccine and BCG-non-vaccinated countries (BCG-). Horizontal lines represent the means.
